# Efficacy of kangaroo mother care combined with neonatal phototherapy in newborns with non-pathological jaundice: A meta-analysis

**DOI:** 10.3389/fped.2023.1098143

**Published:** 2023-01-25

**Authors:** Xiang Huang, Meiling Chen, Rongrong Fu, Wei He, Yujing He, Haojie Shentu, Suping Zhu

**Affiliations:** ^1^ Department of Pediatric, Ningbo Yinzhou No. 2 Hospital, Ningbo, China; ^2^ The Public Health College, Zhejiang Chinese Medical University, Hangzhou, China; ^3^ The First Clinical Medical College, Zhejiang Chinese Medical University, Hangzhou, China; ^4^ State Key Laboratory Breeding Base of Green Chemistry Synthesis Technology, Zhejiang University of Technology, Hangzhou, China; ^5^ The Second Clinical Medical College, Zhejiang Chinese Medical University, Hangzhou, China; ^6^ The Medical Imaging College, Hangzhou Medical College, Hangzhou, China

**Keywords:** kangaroo-Mother care method, phototherapy, skin-to-skin contact, hyperbilirubinemia, infant, meta—analysis

## Abstract

**Background:**

The kangaroo-mother care method (KMC) is a skin-to-skin contact-centered care approach with numerous benefits for neonates, but its impact on the treatment of jaundiced neonates is unknown. This study aimed to investigate the efficacy of KMC combined with neonatal phototherapy (NNPT) in treating neonates with non-pathological jaundice.

**Methods:**

Relevant articles were searched in PubMed, Embase, Web of Science, and Cochrane Library databases from database establishment to April 2022. The outcomes included, without limitation, serum bilirubin levels, and duration of phototherapy.

**Results:**

This meta-analysis included five studies (4 randomized controlled trials and 1 observational study) involving four hundred eighty-two neonates with non-pathological jaundice. The results showed that the group receiving KMC combined with NNPT had lower serum bilirubin at 72 h after intervention [weighted mean difference (WMD) = −1.51, *p* = 0.03], shorter duration of phototherapy [standard mean difference (SMD) = −1.45, *p* < 0.001] and shorter duration of hospitalization (SMD = −1.32, *p* = 0.002) compared to NNPT group. There was no difference in peak bilirubin in both groups of neonates (WMD = −0.12, *p* = 0.62).

**Conclusions:**

KMC combined with NNPT helped to treat non-pathological jaundice in newborns compared to NNPT alone.

## Introduction

Unconjugated hyperbilirubinemia often occurs in neonates. Jaundice symptoms are often physiological or caused by breastfeeding (1). It affects 85% of neonates (2), and while the prognosis is favorable for most of them, pathological jaundice, bilirubin encephalopathy, hearing loss, and seizures may develop without appropriate monitoring or treatment (3). It is a disease burden in countries of all income levels and often arouses physicians' concerns and parents’ anxiety (4).

Neonatal phototherapy (NNPT), exchange transfusion, and pharmacotherapy are applied to treat neonatal jaundice (5). Since the 1950s, NNPT has been the preferred treatment for neonatal jaundice, as it can reduce serum indirect bilirubin levels, and prevent acute and chronic encephalopathy (6–8). However, some studies showed that NNPT could cause short-term side effects such as heat and water-electrolyte imbalance, bronze baby syndrome, the influence on the retina of the eye (7, 9, 10), and long-term side effects such as tumors (7, 11, 12) and allergic diseases (7, 10).

Kangaroo-mother care method (KMC) was first reported in a Columbia hospital in 1984. KMC entails skin-to-skin care of the neonate in a kangaroo position at or shortly after birth (with the naked infant in a prone position on the mother's exposed chest and abdomen), as well as breastfeeding and close follow-up, as appropriate (13, 14). Studies showed that KMC facilitated a reduction in neonatal illness and the occurrence of nosocomial infections, decreasing mortality in low-birth-weight infants, and lessening the duration of hospital stays and medical costs (13, 15). It has also been reported that jaundiced newborns performing KMC favored a shorter duration of phototherapy (16, 17). To date, no relevant meta-analysis has been found in the database. Therefore, the present study aimed to investigate the efficacy of KMC combined with NNPT in treating neonatal non-pathological jaundice.

## Method

### Search strategy

This meta-analysis was directed by recommendations of the Preferred Reporting Items for Systematic Reviews and Meta-Analyses (PRISMA) (18). Two researchers systematically retrieved electronic databases (PubMed, Embase, Web of Science, and Cochrane Library databases) using prearranged terms from database inception to April 2022 and primarily searched for articles related to KMC intervention for neonatal jaundice. The “PICOS” principles (Participant, Intervention, Comparison, Outcome, Study design) were used throughout the meta-analysis. The entire formula used for searching was as follows: (“Infant, Newborn” [Mesh] OR “Infants, Newborn” OR “Newborn Infant” OR “Newborn Infants” OR “Newborns” OR “Newborn” OR “Neonate” OR “Neonates” OR “Infant” [Mesh] OR “Infants”) AND (“Jaundice” [Mesh] OR “Icterus” OR “Hyperbilirubinemias” OR “Bilirubinemia” OR “Bilirubinemias” OR “Hyperbilirubinemia” [Mesh]) AND (“Kangaroo-Mother Care Method” [Mesh] OR “Care Method, Kangaroo-Mother” OR “Care Methods, Kangaroo-Mother” OR “Kangaroo Mother Care Method” OR “Kangaroo-Mother Care Methods” OR “Method, Kangaroo-Mother Care” OR “Methods, Kangaroo-Mother Care” OR “Kangaroo Mother Care” OR “Care, Kangaroo Mother” OR “Kangaroo-Mother Care” OR “Care, Kangaroo-Mother” OR “Skin-to-skin contact” OR “KMC” OR “SSC”). In addition, we reviewed the reference lists of the retrieved articles to identify more relevant studies. The Prospero registration number for this meta-analysis was CRD42022344508.

### Inclusion and exclusion criteria

The studies included met the following criteria: (1) the original research was a randomized controlled trial (RCT) or observational study. (2) The study population was non-pathologically jaundiced newborns. (3) The intervention group used KMC based on NNPT. (4) Control group had NNPT without KMC. (5) Articles published in English.

The exclusion criteria were as follows: (1) relevant or detailed data could not be extracted, or the article could not be integrated with data from other articles. (2) The study was still in the protocol stage or was ongoing, or the full text was unavailable. (3) The study was from the same group of participants. The latest or most complete research was included as the article is continuously updated.

### Indicator measurement

KMC may encompass a range of mother-to-infant interventions, primarily referring to skin-to-skin contact between mother and infant (14). NNPT is a method of treating neonatal jaundice by irradiating the skin to lower serum bilirubin levels. During NNPT, the baby is naked except for the eyes and genitals.

### Data extraction and quality evaluation

Two independent researchers extracted data from articles that met the inclusion criteria using a predesigned data extraction form and assessed study quality. When disagreements arose, a consensus was completed with a third researcher. The following information was extracted: authors, year of publication, country, study design, the onset of jaundice, the sample size of intervention and control groups, KMC usage frequency, feeding patterns, gestational age, weight, outcome indicators, and inclusion and exclusion criteria for neonatal recruitment in studies.

The methodological quality of RCT was assessed using the Cochrane Collaboration Risk of Bias Assessment Tool (19), categorized as low risk, high risk, or unclear risk. The quality of observational studies was assessed using the Newcastle-Ottawa Quality Assessment Scale (NOS) with a maximum score of 9, and articles with high study quality scored 6–9 (20). The evaluation results were displayed in the form of graphs or charts.

### Statistical analysis

The extracted data were analyzed by Review Manager 5.3 analysis software. Continuous variables were evaluated by weighted mean difference (WMD) or standardized mean difference (SMD) and 95% confidence interval (CI). WMD truly reflected the trial effect in original units, and SMD was suitable for pooled analysis of data with different units or large differences in means. A value of *p* < 0.05 was statistically significant. The X^2^ test was primarily used to detect the heterogeneity in the study, which was quantified using the *I*^2^ statistic (21). As *I*^2 ^≥ 50%, heterogeneity was considered statistically significant, and a random-effects model was used; otherwise, a fixed-effects model was used (22, 23). Funnel plots were used to test for publication bias or other biases (24, 25). When the number of included original studies was more than ten (26), the stability of the results could be tested by excluding literature on a case-by-case basis for sensitivity analysis.

## Results

### Literature retrieval

**Figure 1:**
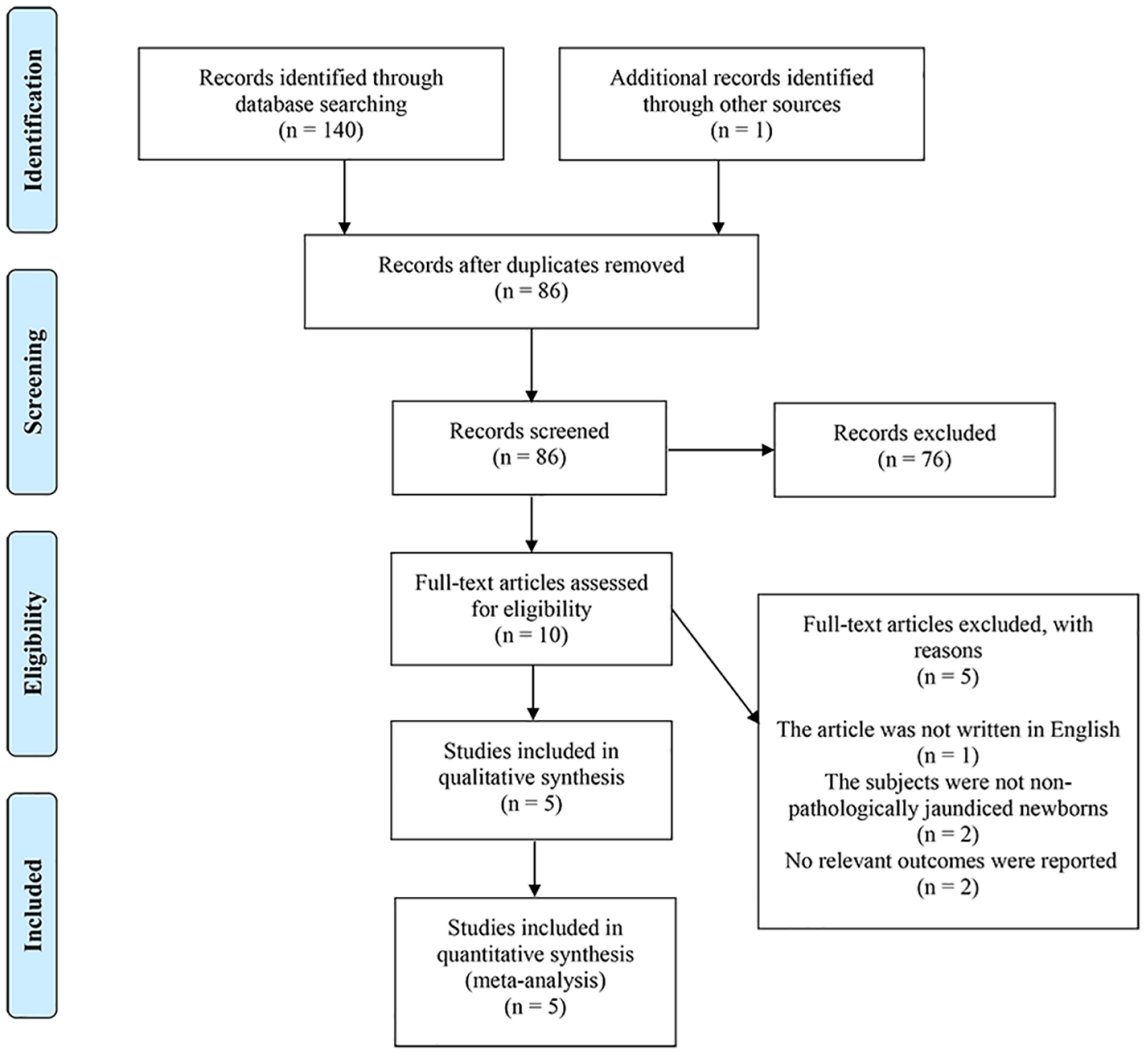
PRISMA retrieval procedures flow chart.

A total of 140 relevant articles were retrieved from electronic databases, and one relevant article was included in the reference list after review. After removing duplicate research papers, there were 86 articles left, and 76 were excluded by reading the titles and abstracts; the remaining ten articles were obtained in full and reviewed. Five of these articles were excluded for the following reasons: articles were published in a language other than English (*n* = 1). The study population was not non-pathologically jaundiced neonates (*n* = 2). Relevant data were unavailable for the article (*n* = 2). Finally, the rest five articles were included in this meta-analysis (16, 17, 27–29). A detailed PRISMA flowchart of the detailed search process and reasons for exclusion is displayed in Figure 1.

### Study characteristics and quality assessment

The characteristics of all articles included in the meta-analysis are listed in Table 1. Five articles were selected, including four RCTs (17, 27–29) and one prospective observational study (16), conducted in different countries: two in Iran (27, 29), one in India (17), one in China (28), and one study in Egypt (16). The analysis included four hundred eighty-two neonates with non-pathological jaundice, with two hundred forty-eight in the intervention group and two hundred thirty-four in the control group. The intervention was KMC combined with NNPT, and the control measure was NNPT. Information on the recruitment criteria for neonates of original studies is presented in Supplementary Table S1.

**Table 1:** Characteristics of included studies in the meta-analysis.

Author, year	Country	Design	Onset of jaundice		KMC + NNPT	NNPT	Time and dose	Feeding patterns
			KMC + NNPT	NNPT				
Jajoo 2022	India	RCT	3.68 ± 1.82 d	4.3 ± 1.8 d	*N* = 25	*N* = 25	1 h, q8h	Enteral/parenteral nutrition
Larma’i 2016	Iran	RCT	–	–	*N* = 53	*N* = 53	30–45 min, ≥ 6t/d a.m. & *p*.m.	Breastfeeding
Li 2017	China	RCT	71.11 ± 15.79 h	72.64 ± 16.84 h	*N* = 112	*N* = 104	≥1 h, tid	–
Lori Kenari 2020	Iran	RCT	–	–	*N* = 30	*N* = 30	30 min, ≥ 5t/d (q3h) a.m. & *p*.m.	Breastfeeding
Samra 2012	Egypt	Prospective Observational Study	66.07 ± 17.7 h	64.32 ± 17.54 h	*N* = 28	*N* = 22	≥1 h, tid	Breastfeeding/feed formula/feed mother's milk

RCT, randomized controlled trial; KMC, kangaroo-mother care method; NNPT, neonatal phototherapy; q8h, quaque 8 h (every 8 h); 6t/d, 6 times a day; a.m., ante meridiem (after midnight); p.m., post meridiem (afternoon + evening); tid, ter in die (3 times a day); 5t/d, 5 times a day; q3h, quaque 3 h (every 3 h); &, and; -, data unavailable.

This meta-analysis assessed the quality of all five included studies, and the quality of the four RCTs was appraised using the Cochrane Collaboration Risk of Bias Assessment Tool. Three studies were assessed as an unclear risk due to insufficient information on the generated sequences in random sequence generation, and the remaining one was evaluated as low risk. Regarding allocation concealment, three studies were categorized as unclear risk and one as high risk. About implementation blinding, three studies were judged as high risk and one as low risk. Four studies were rated as low risk concerning incomplete outcome data, selective outcome reporting, and free of other biases. NOS was used to rate the quality of one observational study with a score of seven. Detailed quality assessment results are presented in Supplementary Table S2.

### Serum bilirubin at 72 h post-intervention

**Figure 2:**
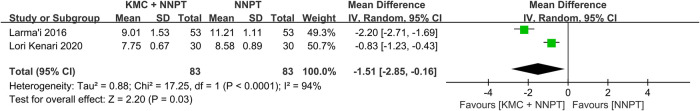
Forest plot comparing KMC + NNPT with NNPT alone on serum bilirubin at 72 h after intervention in neonates with non-pathological jaundice.

Serum bilirubin levels after 72 h in participants after KMC combined with NNPT and NNPT alone were provided by two studies (27, 29), with eighty-three neonates in each group. Neonates receiving KMC combined with NNPT had lower serum bilirubin at 72 h post-intervention than controls (WMD = −1.51, 95%CI: (−2.85)–(−0.16), *p* = 0.03), (Figure 2). It indicated that KMC combined with NNPT contributed to a decrease in serum bilirubin 72 h after the intervention compared to the control group.

### Peak bilirubin

**Figure 3:**
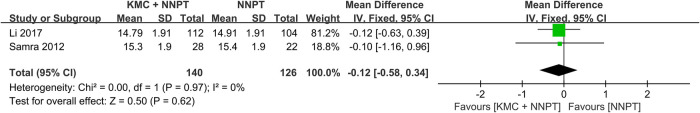
Forest plot comparing KMC + NNPT with NNPT alone on peak bilirubin in neonates with non-pathological jaundice.

The effect of KMC combined with NNPT vs. NNPT alone on peak bilirubin in neonates with non-pathological jaundice was reported in two studies (16, 28). It was found that in the intervention group (one hundred forty) and in the control group (one hundred twenty-six) showed homogeneity between studies with no statistical difference between both groups (WMD = −0.12, 95%CI: (−0.58)–0.34, *p* = 0.62, *I*^2^ = 0%) (Figure 3).

### Duration of phototherapy

**Figure 4:**
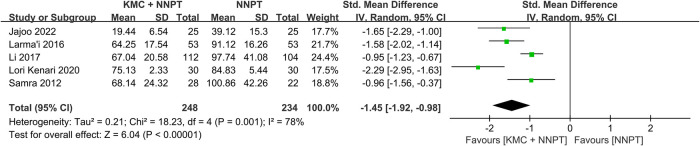
Forest plot comparing KMC + NNPT with NNPT alone on the duration of phototherapy in neonates with non-pathological jaundice.

The duration of phototherapy in neonates with non-pathological jaundice in the KMC combined with NNPT group (two hundred forty-eight) and in the control group (two hundred thirty-four) was reported in five studies (16, 17, 27–29). It was found that neonates who received KMC combined with NNPT had a shorter duration of phototherapy than the NNPT alone group. However, there was relatively large heterogeneity between the included studies (SMD = −1.45, 95%CI: (−1.92)–(−0.98), *p* < 0.001, *I*^2^ = 78%) (Figure 4). It showed that KMC combined with NNPT helped reduce phototherapy duration.

### Duration of hospitalization

**Figure 5:**
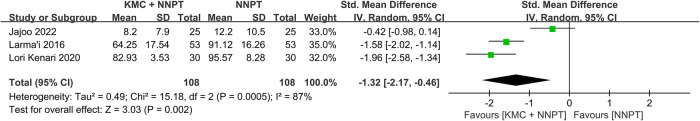
Forest plot comparing KMC + NNPT with NNPT alone on the duration of hospitalization in neonates with non-pathological jaundice.

According to statistics from three studies (one hundred eight neonates in each group) (17, 27, 29), who received KMC combined with NNPT spent less time in the hospital than control (SMD = −1.32, 95%CI: (−2.17)–(−0.46), *p* = 0.002) (Figure 5). It demonstrated that the intervention facilitated a shorter duration of hospitalization for neonates with non-pathological jaundice.

### Publication bias

The presence of publication bias was tested by funnel plot. Compared with the control group, the funnel plot of KMC combined with NNPT on the duration of phototherapy showed a roughly symmetrical inverted funnel shape indicating no apparent publication bias (Supplementary Figure S1).

## Discussion

Jaundice may occur in neonates for the following reasons, (1) high number and short life span of red blood cells in neonates (30). (2) Low levels of bindable proteins in hepatocytes (31). (3) Low catalytic activity of uridine diphosphate glucuronosyltransferase (UDPGT) (31). (4) Higher β-glucuronidase in the neonatal intestine increases the hydrolysis of bound bilirubin (32). (5) Neonatal bilirubin is less stable and easily hydrolyzed in the intestine and transported back to the liver and circulatory system *via* enterohepatic circulation (31).

Initially, NNPT was used to treat unconjugated hyperbilirubinemia to reduce the use of blood exchange therapy. The mechanism of NNPT action lies in the conversion of naturally toxic nonpolar Z, Z-bilirubin into polar and water-soluble photoisomerization products as well as structural isomers (30, 33). The irreversibly formed structural isomer lumirubin (30) has greater solubility and a short half-life in plasma (34). It is readily excreted from urine and bile (30). It was considered the main mechanism (34). In addition, a small fraction of bilirubin is converted into products excreted through urine by degradation (35) or photooxidation (30, 36).

NNPT using visible light to treat neonatal unconjugated hyperbilirubinemia is often considered an inexpensive, simple, most used, and relatively safe treatment option (30, 33, 37). However, there are some short- or long-term side effects of NNPT (33), such as damage to the erythrocyte membrane and bronchial and pulmonary dysplasia (38). As Bergman (39) pointed out, “skin-to-skin contact is the natural “habitat’ for human infants.” When this contact is hindered, the newborn will cry. Most notably, the use of NNPT alone separates the mother from the infant, whereas the combination of skin-to-skin contact care meets the emotional needs of the mother and infant (40) and reduces neonatal stress response (41). For decades, KMC has been regarded as a resultful, noninvasive, simple, and cost-effective care method, particularly when used in low-resource settings in developing countries (42–45). KMC has multiple benefits for neonates in the short or long term, such as promoting breastfeeding (45, 46), stabilizing neonatal physiology (47), and regulating microbiota (48).

The mechanisms by which KMC is effective in jaundiced newborns include the direct effect of the intervention on the newborn, the feedback effect of maternal benefit, and other benefits to the newborn. Firstly, babies who receive KMC develop specific behavioral patterns, such as “crawling” to their mother's nipples (49, 50), and the mother's lactation reflex is enhanced, resulting in increased milk production (49). Consequently, KMC contributed to breastfeeding (13, 51–53). Breastfeeding reduced the enterohepatic circulation of bilirubin and lowered serum bilirubin levels *via* the three pathways described below. (1) Breastfeeding increased neonatal feeding, providing adequate nutrition and calories to babies (54), allowing them to gain body weight (49, 54), improving intestinal motility, and accelerating meconium excretion (55, 56). Meconium excretion was associated with unconjugated bilirubin levels (54) and bilirubin enterohepatic circulation (55, 56). Additionally, increased caloric intake helped reduce the release of fatty acids that interfered with the formation of conjugated bilirubin (57) or favored UDPGT production (58), lowering serum bilirubin concentration. (2) Breastfeeding decreased supplemental formula, and therefore water intake was reduced (55, 59). Therefore, the enterohepatic circulation of bilirubin was decreased, and jaundice was also improved. (3) Breastfeeding led to earlier maturation of bilirubin-binding enzymes in the liver of babies (16, 60). Apart from breastfeeding reduced enterohepatic circulation of serum bilirubin through various pathways, neonates receiving KMC lay prone on the mother's chest and abdomen, and the vibrations generated by the contact accelerated intestinal excretion, which contributed to bilirubin elimination from the body *via* the digestive pathway (16, 27). Thus, serum bilirubin levels were reduced. The decrease in serum bilirubin at 72 h after KMC intervention could be explained by the decrease in serum bilirubin levels.

Simultaneously, the benefits of KMC for the mother are fed back to the newborn, assisting the neonate in reducing the degree of jaundice and resulting in a virtuous circle. This study would describe three parts: the mother's emotional, vagal, and hormonal regulation. (1) KMC shortened mother-infant separation time due to NNPT, improving the mother's mood (61), promoting breastfeeding (62), and helping the neonate recover from the jaundice state. (2) Frequent touching of the newborn by mother receiving KMC stimulated the vagus nerve of the newborn (63) and promoted gastric motility, which helps to lower serum bilirubin (64), thereby shortening the length of hospital stay. (3) KMC regulated neonatal-related hormone levels synergistically by regulating the mother's endocrine system. On the one hand, salivary cortisol is a stress hormone released by the hypothalamic-pituitary-adrenal axis (65, 66). KMC reduced salivary cortisol levels in neonates (51, 67), improving sleep-wake cycles (51). On the other hand, KMC synergistically promoted the synthesis of oxytocin in the hypothalamus of mothers and infants (67). It is important in reducing maternal stress anxiety and neonatal stress response, enabling positive maternal-infant interactions, and promoting breastfeeding (68–70). Apart from this, increased oxytocin release raised the temperature at the mother's breast, promoting a synergistic regulation of the mother's body temperature and the heat requirements of the newborn, which facilitated the maintenance of body temperature stability (51, 71).

Finally, other effects of KMC on the baby can also impact the recovery of jaundiced newborns. (1) KMC alleviated neonatal pain (72, 73). (2) KMC reduced the amount of energy consumed by newborns due to crying (51, 74), so more energy was available for increased bowel movements, weight gain, and health recovery. All of this contributed to lower neonatal serum bilirubin levels and shortened the duration of phototherapy and hospital stay.

The cessation time of NNPT in the included studies was defined according to a decline in serum bilirubin to certain levels. The findings of this study revealed that the serum bilirubin level was lower in the KMC group after 72 h of intervention than in the control group. Therefore, the time required by NNPT to reduce the serum bilirubin levels to the corresponding threshold was also shorter in the KMC group, which might explain the shorter duration of phototherapy in the KMC group. Most included studies were neonates admitted for jaundice (16, 27, 29), and the main treatment objective was to improve jaundice and reduce serum bilirubin levels (75). In the KMC group, there was an advantage in the shortening of hospital stays due to lower serum bilirubin levels and less duration of phototherapy. Less time spent in the hospital helped to increase mother-infant interaction, reducing the financial burden on families and lower medical costs (76). Peak bilirubin is the highest value of total serum bilirubin (77). This study showed no difference in peak bilirubin between the two groups, and the mechanism needs further investigation.

Since the outbreak of coronavirus disease 2019 (COVID-19), the epidemic has spread rapidly among people in close contact (78), so methods of separating people from each other, such as wearing masks and drawing one-meter lines, have become popular. However, this posed a challenge to maternity (79), which might mean that mothers could not be in close contact with their newborns, adversely affecting both mother and baby. If COVID-19 is suspected or diagnosed, the World Health Organization recommends that mothers and infants could use skin-to-skin contact and other measures (80). However, there was no evidence of vertical transmission from a coronavirus-infected mother (81), and increased virus transmission was not observed in newborns receiving skin-to-skin care (82). As a result, the World Health Organization recommendations for neonatal skin care could be followed (80).

As far as we know, no previous meta-analyses have been conducted to evaluate the effect of KMC combined with NNPT on outcomes in jaundiced neonates. Only one systematic review (76) of qualitative analyses reported changes in bilirubin levels and phototherapy duration. We are the first meta-analysis comparing KMC combined with NNPT vs. NNPT alone for neonatal jaundice in strict accordance with PRISMA guidelines.

Although the present meta-analysis generated objective results, there were some limitations. The first was the limited number of studies that could be included, which prevented our study from determining the effect of KMC on other outcomes, such as skin bilirubin. Secondly, some studies reported that in breastfed newborns, proper feeding can alleviate jaundice (60). Feeding patterns was correlated with neonatal jaundice, but there are limited data related to feeding patterns. Therefore, subgroup analysis could not be done in this study to explore its effect on jaundice. In addition, the design of the included studies prevented us from exploring the role of KMC on different types of jaundice in newborns. Finally, the overall findings should be treated with caution because the small number of studies, as well as the different sample sizes, design methods, and confounding factors of different studies, made this study somewhat heterogeneous, resulting in a decrease in credibility inevitably.

In conclusion, KMC combined with NNPT was more effective than NNPT alone in treating non-pathological jaundice in newborns. KMC combined with NNPT reduced serum bilirubin at 72 h after intervention in jaundiced neonates and shortened the duration of phototherapy and hospitalization. However, there was no apparent benefit of the intervention in peak bilirubin. Additional large RCTs are required to provide more data to validate the findings of this study.

## Data Availability

The original contributions presented in the study are included in the article/**Supplementary Material**, further inquiries can be directed to the corresponding author/s.
