# Supplementary material for: Efficacy of kangaroo mother care combined with neonatal phototherapy in newborns with non-pathological jaundice: A meta-analysis
Source: Front Pediatr. 2023 Jan 25;11:1098143. doi: 10.3389/fped.2023.1098143 (PMC10112003; doi:10.3389/fped.2023.1098143)
Supplement: Supplementary file 1 [file Table1.docx]

**Supplementary Table 1.** Characteristics of included studies in the meta-analysis.

| Author, year | GA and Weight | Type of jaundice | Instrument | Outcome | Inclusion criteria | Exclusion criteria |
| --- | --- | --- | --- | --- | --- | --- |
| Jajoo 2022 | 30–40 wk  1001–1999 g | Physiological | Seefar | Duration of phototherapy  Duration of hospital stay  Rate of fall of bilirubin | - | Onset of jaundice in the first 24 h; neonates with gross congenital anomalies; exchange transfusion; hemodynamically unstable; rapidly increasing serum bilirubin (<5 mg/dL/d); requiring continuous positive airway pressure |
| Larma'i 2016 | - | Physiological | XHZ–90  (David, China) & Five lamps | Duration of phototherapy  Duration of hospital stay  Serum bilirubin | - | Onset of jaundice in the first 24 h; breastfeeding jaundice; blood group incompatibility; positive Coombs’ test results; hemolysis |
| Li 2017 | 35–40 wk  2000–3750 g | Physiological/breastfeeding-associated | YG–I & XHZ (David, China) | Duration of phototherapy  Peak bilirubin | Onset of jaundice at 48–72 h of age; peak serum bilirubin in 3–5 days; the rate of rising of bilirubin <5 mg/dl/d; conjugated bilirubin level <2 mg/dl at any time | Onset of jaundice in the first 24 h; prolonged jaundice persisting beyond the third week of life; breastmilk jaundice; late onset of clinical jaundice (5–10 days of age); neonates with small GA or extremely low birth weight; exchange transfusion; presence of known cause and/or risk factor for hyper-bilirubinemia; babies with peak serum bilirubin level appear at day 15; rising rate of bilirubin over 1–2 mg/dL/d; due to maternal causes |
| Lori Kenari 2020 | 37–42 wk  - | Physiological | Tucson model 022 (Iran) & Eight lamps (Philips TL 20W/52 Germany) | Duration of phototherapy  Duration of hospital stay  Serum bilirubin | All breastfed infants older than 48 h of age with bilirubin level of 15–20 mg/dl; lack of blood exchange transfusion, gestational age of 37–42 wk; not taking bilirubin-lowering drugs; without congenital and genetic abnormalities | Onset of jaundice in the first 24 h; breastfeeding jaundice; blood group incompatibility; positive Coombs’ test results; hemolysis; direct hyperbilirubinemia; bile duct and digestive tract obstruction; lack of glucose–6–phosphate dehydrogenase; due to maternal causes |
| Samra 2012 | 35–40 wk  2000–3750 g | Physiological/breastfeeding-associated | Mechanizmi (Slovenia) & Six lamps (Atom phototherapy SL 20 S.B W–NU) | Duration of phototherapy  Peak bilirubin | Onset of jaundice at 48–72 h of age; peak serum bilirubin in 3–5 days; the rate of rising of bilirubin <5 mg/dl/d; conjugated bilirubin level <2 mg/dl at any time | Onset of jaundice in the first 24 h; prolonged jaundice persisting beyond the third week of life; late onset of clinical jaundice (5–10 days of age); neonates with small GA or extremely low birth weight; exchange transfusion; presence of known cause and/or risk factor for hyperbilirubinemia; neonates with congenital anomalies; due to maternal causes |

GA, gestational age; wk, weeks; &, and; -, data unavailable.
