# Supplementary material for: Efficacy of kangaroo mother care combined with neonatal phototherapy in newborns with non-pathological jaundice: A meta-analysis
Source: Front Pediatr. 2023 Jan 25;11:1098143. doi: 10.3389/fped.2023.1098143 (PMC10112003; doi:10.3389/fped.2023.1098143)
Supplement: Supplementary file 2 [file Table2.doc]

**Supplementary Table 2.** Quality assessment of studies included.

| Author, year,  Study (RCT) | Sequence  Generation | Allocation  Concealment | Blinding | Incomplete  outcome data | Selective  outcome reporting | Free of  other bias |
| --- | --- | --- | --- | --- | --- | --- |
| Jajoo 2022 | unclear risk | unclear risk | high risk | low risk | low risk | low risk |
| Larma'i 2016 | unclear risk | unclear risk | high risk | low risk | low risk | low risk |
| Li 2017 | unclear risk | unclear risk | high risk | low risk | low risk | low risk |
| Lori Kenari 2020 | low risk | high risk | low risk | low risk | low risk | low risk |

| Author, year,  Study (Observational Study) | **Selection (Out of 4)** | | | | **Comparability**  **(Out of 2)** | **Outcomes (Out of 3)** | | | **Total**  **(Out of 9)** |
| --- | --- | --- | --- | --- | --- | --- | --- | --- | --- |
|  | Representativeness of exposed cohort | Selection of nonexposed cohort | Ascertainment  of exposure | Outcome not present at the start of the study |  | Assessment of outcomes | Length of follow-up | Adequacy of follow-up |  |
| Samra 2012 | 1 | 1 | 1 | 1 | 2 | 1 | 0 | 0 | 7 |

The RCTs and observational studies were assessed by the Cochrane Collaboration’s tool and Newcastle-Ottawa Quality Assessment Scale, respectively.

Risk of bias was assessed as “low risk”, “high risk” or “unclear risk”.
