# Supplementary figures and images for: Efficacy of kangaroo mother care combined with neonatal phototherapy in newborns with non-pathological jaundice: A meta-analysis
Source: Front Pediatr. 2023 Jan 25;11:1098143. doi: 10.3389/fped.2023.1098143 (PMC10112003; doi:10.3389/fped.2023.1098143)

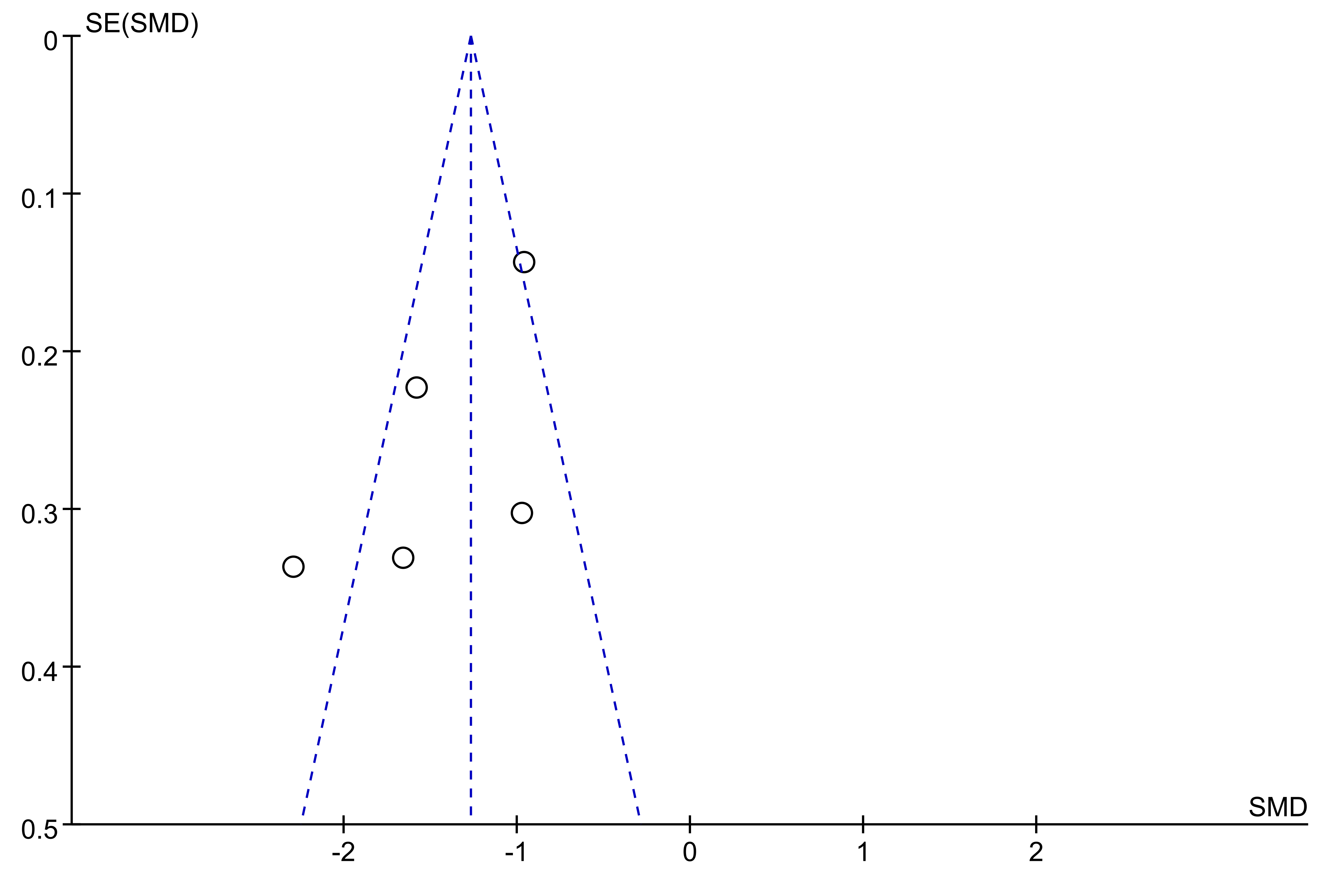

Supplement: Supplementary file 3 [file Image1.tif]
